# Localized zinc distribution in shark vertebrae suggests differential deposition during ontogeny and across vertebral structures

**DOI:** 10.1371/journal.pone.0190927

**Published:** 2018-01-11

**Authors:** Vincent Raoult, Nicholas Howell, David Zahra, Victor M. Peddemors, Daryl L. Howard, Martin D. de Jonge, Benjamin L. Buchan, Jane E. Williamson

**Affiliations:** 1 Department of Biological Sciences, Macquarie University, Sydney, NSW, Australia; 2 ANSTO, Lucas Heights, NSW, Australia; 3 NSW DPI Fisheries, Sydney Institute of Marine Science, Mosman, NSW, Australia; 4 Australian Synchrotron, Clayton, Victoria, Australia; Department of Agriculture and Water Resources, AUSTRALIA

## Abstract

The development of shark vertebrae and the possible drivers of inter- and intra-specific differences in vertebral structure are poorly understood. Shark vertebrae are used to examine life-history traits related to trophic ecology, movement patterns, and the management of fisheries; a better understanding of their development would be beneficial to many fields of research that rely on these calcified structures. This study used Scanning X-ray Fluorescence Microscopy to observe zinc distribution within vertebrae of ten shark species from five different orders. Zinc was mostly localised within the *intermedialis* and was generally detected at levels an order of magnitude lower in the *corpus calcareum*. In most species, zinc concentrations were higher pre-birth mark, indicating a high rate of pre-natal zinc deposition. These results suggest there are inter-specific differences in elemental deposition within vertebrae. Since the deposition of zinc is physiologically-driven, these differences suggest that the processes of growth and deposition are potentially different in the *intermedialis* and *corpus calcareum*, and that caution should be taken when extrapolating information such as annual growth bands from one structure to the other. Together these results suggest that the high inter-specific variation in vertebral zinc deposition and associated physiologies may explain the varying effectiveness of ageing methodologies applied to elasmobranch vertebrae.

## Introduction

Large-scale commercial fishing practices have led to global declines in fish stocks and significant shifts in the structure of marine communities [1]. Management efforts have increased proportionally to these declines but are often still marred by a lack of biological information necessary to manage fisheries sustainably or predict recovery of a species [2]. Considering the important role these predators play in ecological stability [3], correct management strategies are crucial [3–5]. Elasmobranchs are particularly vulnerable to overfishing due to their often slow growth rates, low fecundity, and delayed onset of maturity [4, 6].

Determining age at maturity and longevity are two components that are critical for effective fisheries management [7, 8]. In elasmobranchs, age is determined by counting bands on the outer anterior and posterior calcified structures of the vertebrae (*corpus calcareum)* [9] rather than the tissue between two *corpus calcareum*, the *intermedialis* [10]. If bands in the *corpus calcareum* are difficult to discern, common practice is to use the bands on the *intermedialis* as a guide [11]. Although elasmobranch vertebrae have been analysed using this method for over thirty years, an understanding of vertebral development is still deficient. For example, the reasons why some species, especially deep-dwelling sharks, have vertebral bands with poor readability is unknown [12, 13] and discrepancies between validated and non-validated age assessments are not well explained [14]. In addition, the relationship between the development of the *intermedialis* and the *corpus calcareum* is not understood, despite the use of the *intermedialis* as a guide for ageing from the *corpus calcareum*. These knowledge gaps have led to researchers using a variety of preparation techniques that aim to increase the accuracy of age band counting but that regularly give somewhat ‘noisy’ results. Concerns about the validity of age assessments has led to calls for consistent methodologies [15, 16] and a more causal understanding of vertebral growth processes [14]. A greater understanding of vertebral development would allow for simpler, more directed ageing techniques with more accurate results that could potentially be applied more widely with a greater level of confidence.

While shark vertebrae have mainly been used for ageing, they have recently been used to track chemical or elemental variables throughout the life of an animal [17, 18]. For example, tissue taken from successive vertebral bands in White sharks (*Carcharodon carcharias*) have allowed researchers to track isotope ratios and diet shifts during ontogeny [19]. Metals within shark tissue can be used in a similar fashion to track changes in trophic levels, patterns of diet, and pollutants that the individual may have absorbed [20]. One such metal, strontium, has the potential to be used for tracking movements of animals across salinity gradients [21]. Moreover, a number of studies have examined vertebral chemistry of sharks to either understand stock structure [22] or nursery sites [23, 24]. There is a growing interest in the use of elemental techniques on vertebrae to examine various aspects of elasmobranch life-history traits, but there is a surprising lack of understanding in the developmental dynamics of elasmobranch cartilage [17]. Just as incorrectly assuming that vertebral growth bands are always accurate indicators of age can lead to improper management guidelines [14], there is risk associated with inferring movement patterns and population dynamics from vertebral element profiles without an understanding of the physiological processes that govern vertebral development.

Recently, researchers have begun to examine elemental distributions within elasmobranch vertebrae using Laser-Ablation Inductively Coupled Plasma Mass Spectrometry (LA ICPMS) to understand how these distributions are related to age-associated visual banding [17] and environmental parameters [25]. Such studies have focused on the *corpus calcareum*, the outer region of the vertebrae, as these are the more heavily calcified structures that are traditionally used for assessing age [26]. This focus is likely partially driven by the fact that LA ICPMS generally acquires data in linear transects, and as a result elemental distributions within other structures of the vertebrae, namely the *corpus intermedialis*, have previously been unobserved. In addition, the structure of the *intermedialis* appears to vary greatly across species, and in some cases does not appear to form continuously [27, 28]. While sequential transects using LA ICPMS have allowed complete elemental maps of vertebrae to be constructed [17], the process has low throughput (single sample at a time) and a relatively low resolution (50–80μm) that may be unable to resolve elemental variations, especially in species with smaller vertebrae. In contrast, Scanning X-ray Fluorescence Microscopy (SXFM) allows for relatively rapid elemental mapping of numerous whole vertebrae at resolutions below 15μm [21]. Using SXFM to examine elemental distributions across whole vertebrae of numerous species would identify wider patterns of elemental distributions, and directly test the hypothesis that growth of the *intermedialis* is directly related to growth of the *corpus calcareum* [11].

Many elements naturally present in seawater are incorporated into elasmobranch vertebrae during growth, and are often preferentially absorbed instead of calcium [17].The focus of elasmobranch elemental research has generally been on these elements, as they may yield clues into the movement patterns or behaviours of these animals [29]. Unlike other elements, zinc is incorporated into vertebrae as a result of physiological rather than environmentally-driven processes, and in teleost otoliths is trapped in the interstitial spaces of expanding matrices [30]. Zinc can therefore be considered an indirect indicator of physiological processes associated with vertebral development. Research on physiologically-driven zinc incorporation in elasmobranch cartilage may allow researchers to better understand vertebral development, how such processes may affect age banding patterns, and how to interpret environmentally-mediated changes in other elements in vertebrae.

Zinc is a commonly found heavy metal, and concentrations of zinc in marine environments increase with depth and are related to silica levels [31]. Zinc has many known structural and functional biochemical roles in vertebrates [32–34], including a strong link with the development of connective tissues and cartilage [35, 36]. It accumulates in marine organisms and is heavily concentrated within eyes [37]. Recent research in marine bird feathers indicates that absorbance of zinc can vary diurnally and between species [38]. In fish, zinc uptake is linked to diet and bone development [39] and is strongly modulated by the gills [40]. Some studies suggest that zinc deposition is environmentally mediated in fishes [41], and the uptake of zinc has been extensively studied to optimize growth rates in cultured fish [42, 43]. In contrast, most studies on elasmobranchs consider zinc solely as an environmental contaminant [44, 45], rather than for its potential role in physiology or development. To date, only one study has assessed fine-scale zinc distribution or variations in zinc concentration through the life history of elasmobranchs using LA ICPMS [25]. This study focused exclusively on the *corpus calcareum* of the round stingray (*Urobatis halleri*) and found that zinc concentrations were positively correlated to water temperature [46]. However, the structure of elasmobranch vertebrae is highly variable, and these patterns of zinc distribution may not be consistent across elasmobranchs with low rates of calcification.

Due to difficulties associated with accessing elemental detectors and obtaining vertebrae, most elemental analyses of elasmobranch vertebrae have been conducted on single species in isolation [17, 22, 24, 25, 29]. Obtaining elemental maps of zinc in shark vertebrae across multiple orders and individuals would provide a more wholistic understanding of zinc incorporation in elasmobranch vertebrae and, by association, physiological processes that may occur in the different structures within vertebrae. Specifically, the aims of this study were to 1) examine patterns of zinc distribution across whole vertebrae through ontogeny within individuals and among species from different orders, and 2) compare zinc distributions between vertebral structures, specifically the *intermedialis* and *corpus calcareum*. We predicted that there would be inter-specific differences in the patterns of zinc incorporation, and that zinc distribution in the *intermedialis* would correlate with distribution within the *corpus calcareum*. This is the first known study to determine the inter-specific distribution of zinc across whole shark vertebrae with results benefitting fisheries managers and conservationists who regularly use vertebrae for ageing or ecological purposes.

## Materials and methods

Multiple species of sharks were acquired from the south-eastern coast of Australia in the New South Wales Shark Meshing (Bather Protection) Program, and from fishing trawlers based in Sydney and Launceston (NSW and Tasmania, respectively). Species included *Carcharodon carcharias*, *Sphyrna zygaena*, *Heterodontus portusjacksoni*, *Carcharhinus obscurus*, *Carcharhinus limbatus*, *Carcharhinus brevipinna*, *Pristiophorus nudipinnis*, *Pristiophorus cirratus*, *Squatina albipunctata*, and *Squatina australis*. Age was previously determined in Raoult et al. [21] from band counts using conventional microscope examination. Age could not be determined for *Pristiophorus* spp. and *Squatina* spp. because band counts for these species relate to somatic growth rather than age [47], but this did not prevent any samples from being analysed with SXFM. Specimen maturity was determined using clasper calcification or uterus wall thickening [48], or, when the carcass was not available, roughly approximated from the size of the shark using age and growth curves from previous research (e.g. Natanson and Skomal [49]). Individuals were caught as bycatch and were not directly harmed because of this study. The Macquarie University Animal Ethics Committee agreed that ethics approval could be waived, and no further permits were required for this study. Animals varied in their stages of maturity and age. Cervical vertebrae were retrieved from individuals and roughly cleaned of tissue with a sharp knife. Each vertebra was then kept frozen (-20°C) until ready for sectioning.

Each sample was manually cleaned with a sterile scalpel before being sectioned. No chemical agents were used. Samples were sectioned using an Isomet circular saw with a diamond-edged blade. Saggital dorso-ventral sections were made through the centre of the vertebrae. Cuts were roughly 0.6mm in thickness dependent on the calcification thickness (more calcified specimens could be cut thinner). While sectioning residue can leave marks or contamination, any attempt to chemically or physically remove potential contamination may by association add additional contaminants, so no further sample processing was conducted. Sectioned samples were immediately placed on Kapton film and covered with Kapton adhesive tape. This created an airtight seal that would prevent dehydration of the samples that can cause severe tissue warping during the long imaging process, and prevents cross-contamination between vertebral sections. Samples were then placed between two microscope slides to keep them flat during transport to the X-ray Fluorescence Microscopy (XFM) beamline [50] at the Australian Synchrotron in Melbourne.

Samples were attached to polycarbonate frames using clear double-sided tape, with roughly 15–20 samples per frame. Smaller samples that would require greater resolution (e.g. sawsharks and angel sharks) were sampled at a 15 micron measurement interval, while larger samples (e.g. whaler sharks) were sampled at a 25 micron measurement interval. Ideal resolution would be higher, but scanning time is resolution and area dependent. Scanning time for each frame varied between16 and 22 hours per specimen depending on the required sensitivity and the total area covered. X-ray fluorescence data were processed using GeoPIXE [51, 52], which takes input estimates of the specimen composition and thickness to correct for self-absorption of the X-ray fluorescence based on a projected-specimen approximation (that is, assumes that the elements are uniformly distributed in the projection direction). Due to these approximations, the presented maps are a good indicator of the relative elemental distribution, although there may be small artefacts due to tissue thickness and density variation (density variations were found to be insignificant by inspection of the Compton scattering signal). In this style of analysis, small unphysical negative concentration values can occur due to errors in background estimation, but these do not significantly affect the interpretation of relative distribution information.

**Fig 1:**
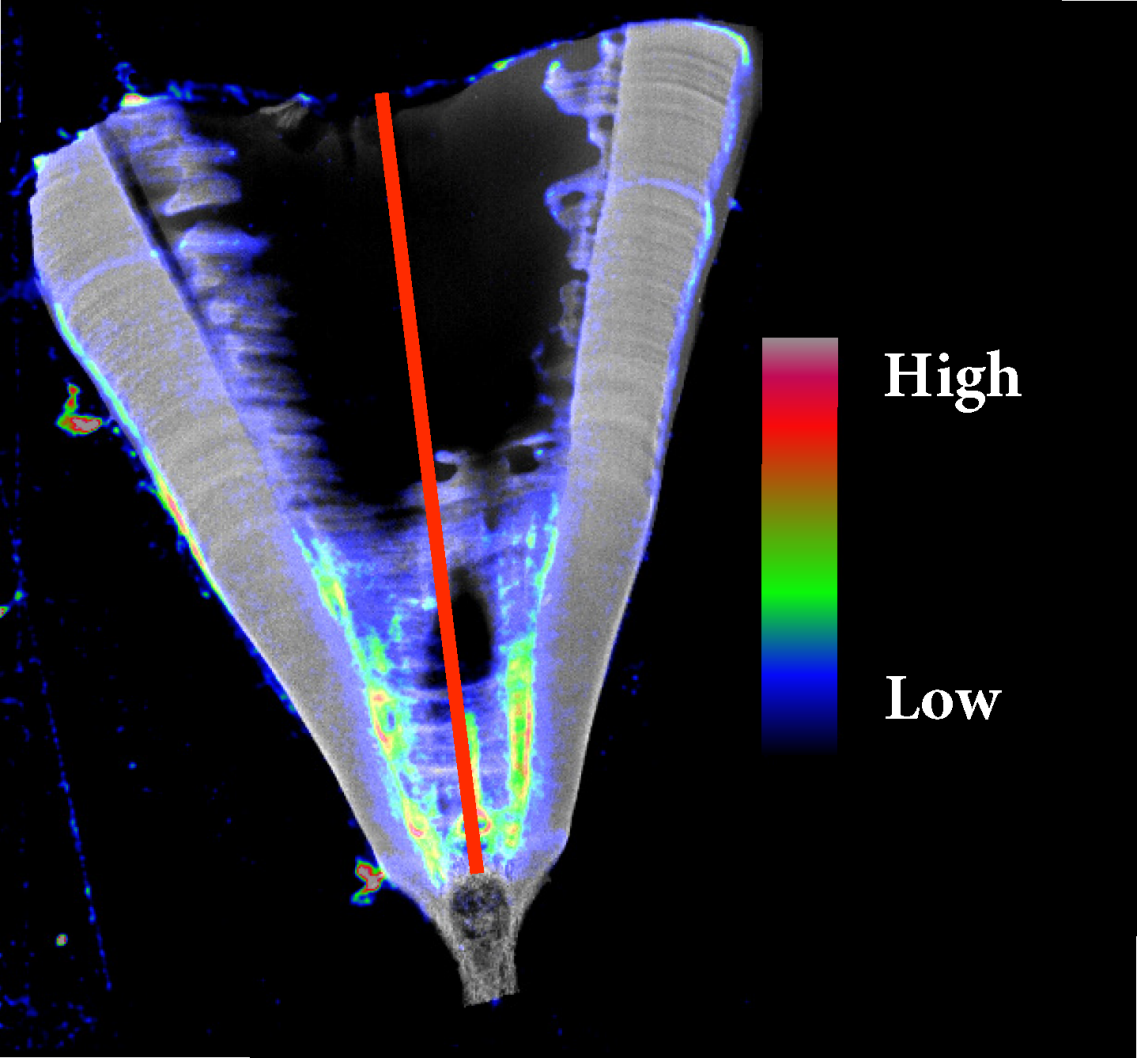
Measured concentrations of zinc in the vertebra of a White shark. Relative zinc concentrations (in colour) obtained from a longitudinal section of a cervical vertebra overlaid onto the Compton Scatter image of a White shark (*Carcharodon carcharias*) with the red line through the *intermedialis* indicating the transect used to extract zinc data for analyses.

To quantify any changes in zinc deposition during ontogeny within an individual, relative structural differences in zinc concentrations were extracted from the larger datasets from a band placed in the *intermedialis* starting from the centre of the vertebrae and projecting outwards (Fig 1). While it is possible to infer absolute concentrations of elements via SXFM, it would require correcting for variations in thickness that may occur across the vertebral sections. In this instance the raw uncorrected data were used, since relative changes across the vertebrae were more informative than absolute concentrations. The focus of this study was to determine relative structural changes in zinc composition, and the high (< 25μm) spatial accuracy of SXFM was more than sufficient to achieve this.

Mean zinc and calcium detections across transects of the *corpus calcareum* in Raoult et al. [21] (which used the same vertebrae) were compared to mean zinc and calcium detections across similar transects in the *intermedialis* to determine whether zinc concentration across vertebral structures differ.

To enable an accurate visual representation of zinc concentrations across the vertebral structure, zinc data were overlaid onto images generated from the Compton scatter (effectively electron/sample density, similar to a generic X-ray) to give the elemental distribution some anatomical context (Fig 1). Birth structures, the vertebral areas that indicate where sharks transitioned from embryos to neonates, were indicated with the presumption that they were characterised by distinct changes in the angle of the *corpus calcareum* [10]. The extracted zinc data were then analysed for every species using a linear regression comparing zinc concentration and distance from the centre of the vertebra (younger age) to the outside of the vertebra (older age) at 15 or 25μm intervals, the acquisition points for the XFM; this led to regressions on roughly 360 ± 20 points per vertebrae on average.

Most studies express zinc concentrations as Zn:Ca ratios [17]. This is done on the basis that elements are incorporated instead of calcium, which is not the case for zinc, and for this reason Zn:Ca ratios may not be an effective way to examine trends in accumulation across species with different rates of calcification and zinc accumulation. For comparative assessment with previous studies, however, we also incorporated relative calcium concentrations into our figures. To make elemental patterns easier to discern, data were analysed using locally weighted smoothing (LOESS) with a span setting of 0.3 provided by the ggplot2 package [53]. All analyses were conducted using R statistical software version 3.3.3 through RStudio [54].

## Results

**Fig 2:**
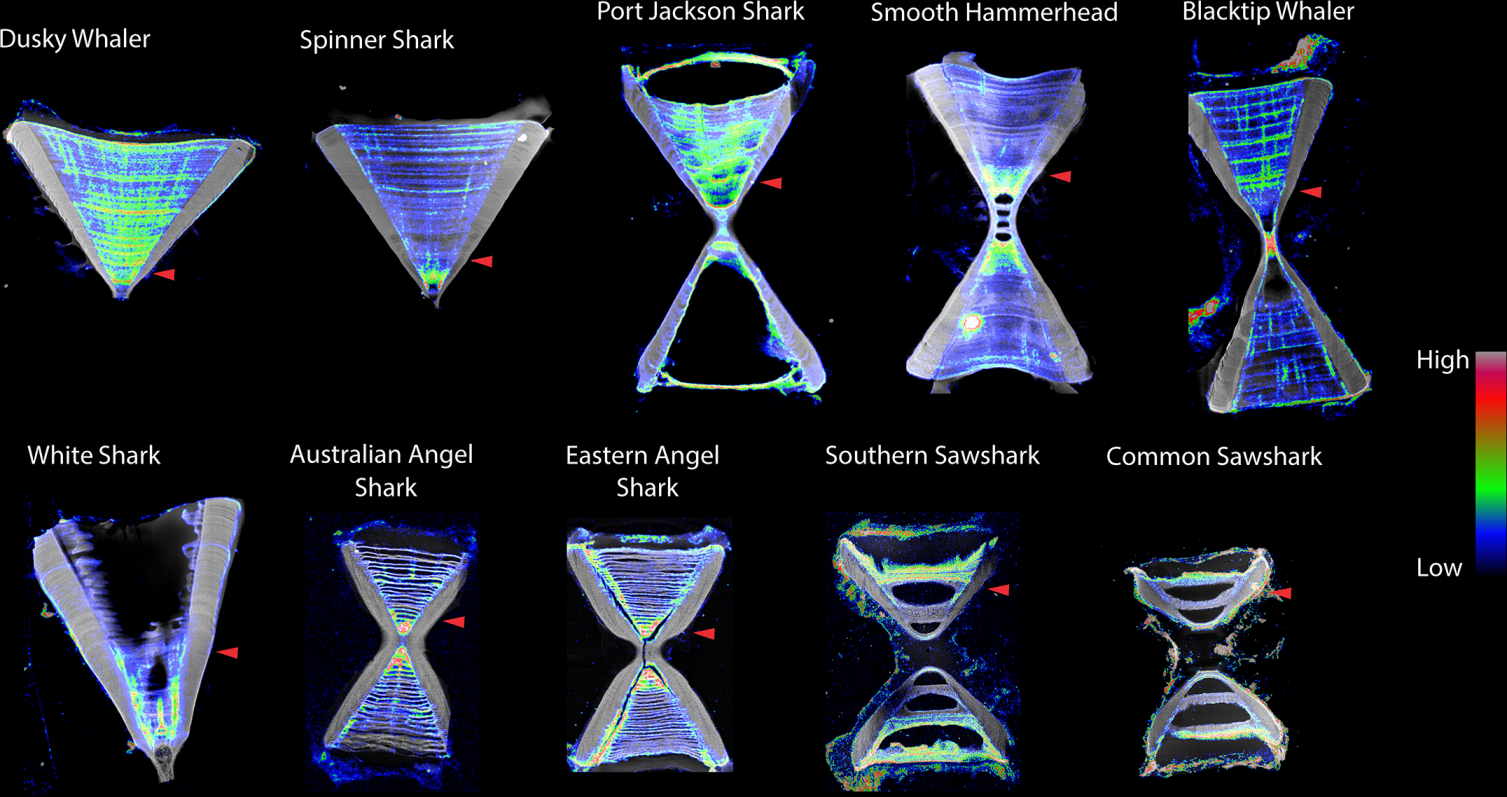
Scanning X-Ray Fluorescence Microscope (SXFM) image of shark vertebrae in the zinc spectrum (size not to scale) for Dusky (*Carcharhinus obscurus*), Spinner (*Carcharhinus brevipenna*), Port Jackson (*Heterodontus portjacksoni*), Smooth Hammerhead (*Sphyrna zygaena)*, Blacktip (*Carcharhinus limbatus)*, White (*Carcharodon carcharias)*, Australian Angel (*Squatina australis*), Eastern Angel (*Squatina albipunctata)*, Southern Sawshark (*Pristiophorus nudipinnis*), and Common Sawshark (*Pristiophorus cirratus)*. Results are overlayed onto Compton scatter maps, essentially an X-ray of the sample (white colour). Colours signify concentrations of zinc. Zinc concentrations are highest in the *intermedialis* in all species, the highest concentrations often occurring pre-birth. Notice the absence of colour (read: zinc) in the *corpus calcareum*, except in the Port Jackson. Birth marks indicated with red arrows.

Zinc was highly localised within the vertebrae of all species of sharks sampled (Table 1, Fig 2). In general, there were lower zinc levels in the *corpus calcareum* of sharks, with the exception of those from the Port Jackson (*Heterodontus portjacksoni*), which displayed zinc banding similar to strontium bands or age bands (Table 1, Fig 2). Mean zinc concentrations were always higher in the *intermedialis* than in the *corpus calcareum*, often by at least an order of magnitude. This difference was much greater than the relative differences in calcium detection in these two structures, which generally showed the opposite pattern of higher detection rates in the *corpus calcareum*, with the exception of Spinner sharks (*Carcharhinus brevipinna*). Zinc concentrations within the *intermedialis* were highly variable between species, and zinc banding within the *intermedialis* appeared to be correlated to age bands in most species.

**Table 1:** Comparison of mean zinc and calcium detection rates (approximate ppm) of line transects across the *corpus calcareum* and the *intermedialis* using SXFM.

Species	Common name	*Corpus calcareum* zinc detection (mean ± S.E.)	*Intermedialis* zinc detection (mean ± S.E.)	*Corpus calcareum* calcium detection (mean ± S.E.)	*Intermedialis* zinc calcium detection (mean ± S.E.)
*Carcharhinus obscurus*	Dusky Whaler	0.35 ± 0.15	18.1 ± 0.39	17,303 ± 113	14,614 ± 285
*Carcharhinus brevipinna*	Spinner	2.97 ± 0.16	23.7 ± 0.46	121,759 ± 1,140	147,552 ± 1,484
*Heterodondus portjacksoni*	Port Jackson	29.99 ± 0.73	51.13 ± 1.97	54,540 ±172	32,950 ± 1,279
*Sphyrna zyaena*	Smooth Hammerhead	8.15 ±0.34	18.6 ± 11.43	53,785 ± 314	29,124 ± 571
*Carcharhinus limbatus*	Blacktip Whaler	-5.69 ± 0.83	12.71 ± 0.78	22,148 ± 287	14,846 ± 670
*Carcharodon carcharias*	White	3.38 ± 0.31	13.39 ± 0.84	38,586 ± 255	16,061 ± 739
*Squatina australis*	Australian Angel	1.1 ± 0.24	13.38 ±0.46	50,114 ± 406	45,709 ± 1,510
*Squatina albipunctata*	Eastern Angel	-1.38 ± 0.30	8.91 ±0.41	233,401 ± 1,120	134,908 ± 3,262
*Pristiophorus nudipinnis*	Southern Sawshark	5.08 ± 1.22	8.44 ± 0.48	190,379 ± 3,471	72,373 ± 3,033
*Pristiophorus cirratus*	Common Sawshark	0.34 ± 1.84	11.37 ± 0.61	113,406 ± 6,454	51,773 ± 3,268

**Fig 3:**
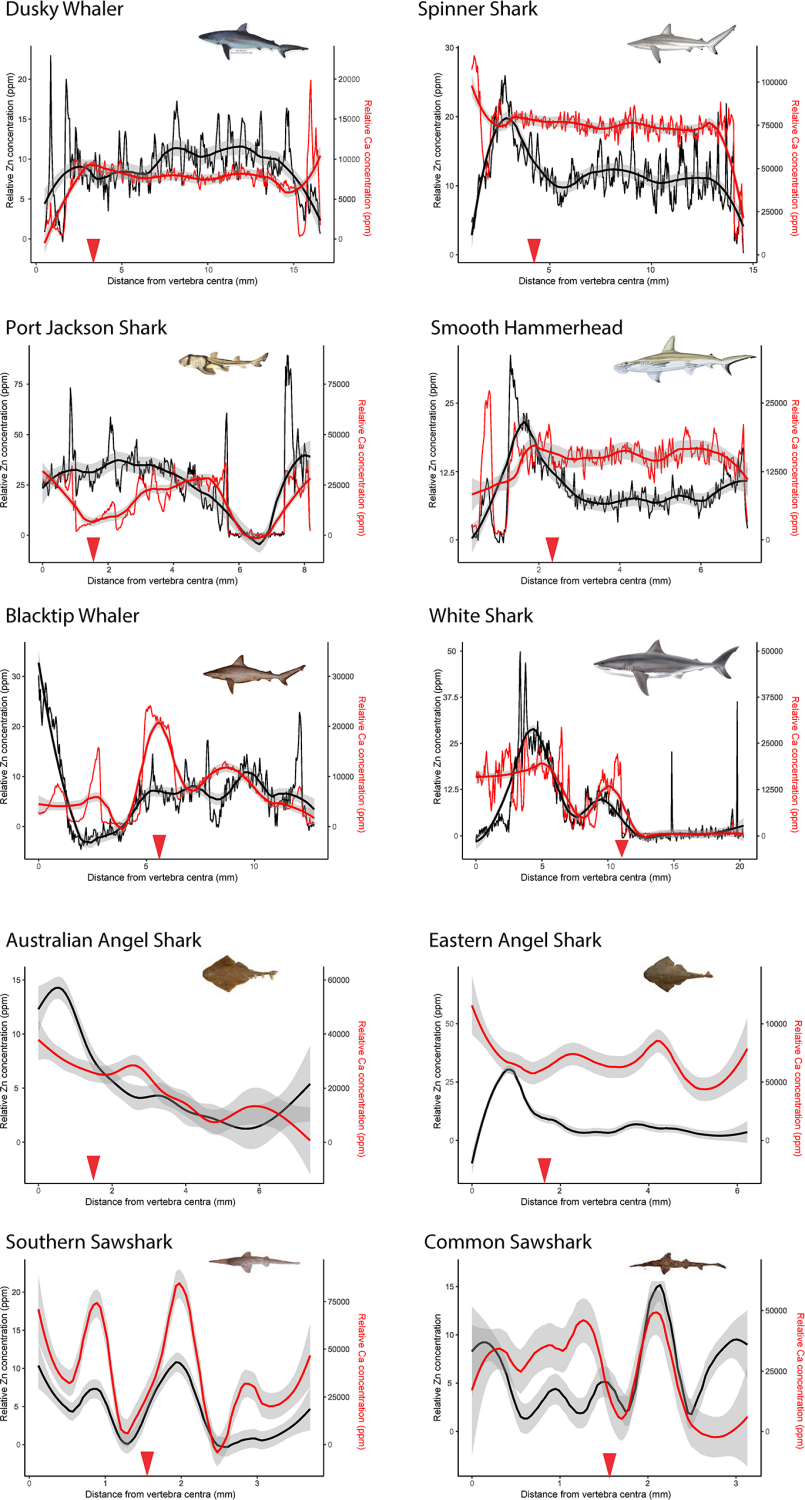
Scatter plots depicting zinc (in black) and calcium (in red) detection rates measured inside vertebral sections of the *intermedialis*, from the centre of the vertebra (distance = 0) to the outside. Raw data with locally-weighed smoothing ± 95% C.I. presented for an individual Dusky (*Carcharhinus obscurus*), Spinner (*Carcharhinus brevipenna*), Port Jackson (*Heterodontus portjacksoni*), Smooth Hammerhead (*Sphyrna zygaena*), Blacktip (*Carcharhinus limbatus*), and White (*Carcharodon carcharias*) sharks. Locally weighed smoothing result summarising trends for Australian Angel (*Squatina australis*, n = 6), Eastern Angel (*Squatina albipunctata*, n = 4), Southern Sawshark (*Pristiophorus nudipinnis*, n = 3), and Common Sawshark (*Pristiophorus cirratus*, n = 3). Approximate locations of birth marks are indicated by red arrows.

While zinc concentrations were highly variable within vertebrae, across individuals, and across species (Fig 3) some patterns were evident. Linear regressions showed significant negative relationships between zinc concentration and distance along the *intermedialis* in 8 of the 10 species examined (Table 2). The exception to this was the Common Sawsharks, which displayed a significantly positive relationship between zinc concentration and distance along the *intermedialis* (Table 2).

**Table 2:** Results of linear regressions of zinc concentrations from the middle of the vertebrae (young age/pre-birth) to the outside of the vertebrae (old age/age at death).

Species	Common name	n	df	F value	Total length (m)	Sex	Age	P value	R^2^	Relationship with age
*Carcharhinus obscurus*	Dusky Whaler	1	320	1.29	2.8	F	21	0.255	N/A	N/A
*Carcharhinus brevipinna*	Spinner	1	333	67.91	2.4	F	12	< 0.001	0.17	Negative
*Heterodondus portjacksoni*	Port Jackson	1	326	33.07	1.15	F	17	< 0.001	0.09	Negative
*Sphyrna zyaena*	Smooth Hammerhead	1	271	5.407	1.7	F	3	0.021	0.015	Negative
*Carcharhinus limbatus*	Blacktip Whaler	1	318	0.05	1.9	F	7	0.81	N/A	N/A
*Carcharodon carcharias*	White	1	506	163.1	2.7	F	9	< 0.001	0.24	Negative
*Squatina australis*	Australian Angel	6	1217	69.54	0.65 ± 0.14	3F, 3M	N/A	< 0.001	0.38	Negative
*Squatina albipunctata*	Eastern Angel	4	772	33.68	1.07 ± 0.07	3F, 1M	N/A	< 0.001	0.23	Negative
*Pristiophorus nudipinnis*	Southern Sawshark	3	668	18.54	0.86 ± 0.15	2F, 1M,	N/A	< 0.001	0.10	Negative
*Pristriophorus cirratus*	Common Sawshark	3	754	10.33	0.71 ±0.09	2M, 1 unknown	N/A	< 0.001	0.06	Positive

For species with multiple samples, total lengths are indicated as means ± S.E. Age was determined using traditional methods in Raoult et al. [21].

## Discussion

Zinc concentrations within and among vertebrae in the species of sharks assessed varied, however, some patterns were evident. Seven of the ten species exhibited substantially higher pre-birth zinc concentrations than in subsequent stages post-birth. Contrary to predictions, zinc was primarily detected in the inner regions of the vertebrae within the *intermedialis*, and was generally detected at levels over an order of magnitude higher than in the *corpus calcareum* for all species other than the Port Jackson. Concentrations of zinc did not correlate to calcium concentrations. Visible bands of zinc that corresponded to traditional visual age bands in the *intermedialis* were observed in most species. This was not the case, however, for sawsharks and angel sharks, in which the *intermedialis* bands were not correlated with age as traditionally measured (see discussions in Raoult [48] and Raoult et al. [21]).

Our study shows that zinc accumulates in shark vertebrae, but the timing and magnitude of accumulation varies within and between species. No single pattern in zinc distribution was evident among all the species tested. Because zinc deposition is driven by physiology [17], the absence of any single pattern suggests that the physiological processes that drive vertebral development are highly varied among elasmobranchs. Zinc deposition in elasmobranchs can be mediated by external temperature [25]. To some degree, this may explain why no single vertebral ageing technique has been widely applicable to a range of species [15]; the development and likely the elemental structure of vertebrae appear to be highly variable within this group. We suggest that a more thorough understanding of vertebral development, and specifically how and why it varies among species of elasmobranchs, would help fisheries managers and researchers understand and predict discrepancies between validated and non-validated ageing methodologies.

The frequent assumption that vertebral growth is generally correlated between the *intermedialis* and the *corpus calcareum* (e.g. in Goldman et al. [11]) requires re-examination when comparing trace concentrations of zinc in these structures. While zinc concentrations in Port Jackson sharks were at similar levels across the vertebral structures and may be correlated, zinc concentrations were either undetectable or orders of magnitude lower in the *corpus calcareum* than in the *intermedialis* in other species, and the difference in calcification between the two structures was much smaller. This elemental relationship appears to be the inverse to that of strontium, which was mainly detected in the *corpus calcareum* in similar species [21]. The cartilage structure of the *intermedialis* is comprised of larger, less densely-packed cells than the *corpus calcareum* [55], and it is possible that this lower level of calcification allows for more zinc to be incorporated into these interstitial spaces. The difference in relative zinc concentration between the two structures is much greater than that of calcium, however, which suggests that calcification rates alone cannot explain differences in zinc concentrations between the structures. These points suggest that elemental deposition, at least for zinc, in the *intermedialis* and the *corpus calcareum* are governed by as yet undetermined different physiological processes.

Seven of the ten species examined had higher zinc concentrations pre-birth than post-birth in the *intermedialis*. Some species of sharks can display maternal signatures [19, 56] across aplacental and placental embryonic development types. For example, muscle and liver tissues from Atlantic Sharpnose neonates have isotope signatures similar to their mother’s [57]. It is thus possible that pre-birth zinc concentrations may be related to the concentrations of zinc that was present in their mother. However, the zinc concentrations in older portions of the vertebrae in this study were generally lower than pre-birth levels. The pattern is further complicated by unexpected patterns of deposition observed in some species with different early developmental strategies. Eastern Angel sharks, which develop aplacentally, had higher pre-birth zinc detection rates than the placental Smooth Hammerhead. Research on a more comprehensive dataset that includes individuals from different generations and locations is needed to determine whether embryonic developmental processes sequester available elements in concentrations related to maternal concentrations, and whether other processes uncouple this relationship during development.

Observed post-birth variations in zinc distributions may be driven by diet or environment [17]. The Common Sawshark is a benthic predator with a diet mainly consisting of invertebrates such as shrimp [58–60]. Decapod shells are known to absorb environmentally available zinc [61], implying that high concentrations of zinc post-birth in this species may, therefore, be related to diet. Conversely, the Southern Sawshark is a piscivorous species that is sympatric with the Common Sawshark over much of its distribution, does not have a similar pattern of zinc deposition, despite feeding at a higher trophic level [58]. If zinc concentrations were related to bioaccumulation, Southern Sawsharks should similarly exhibit higher zinc concentrations with increasing age. Similarly, the lack of a positive relationship between zinc concentrations and distance along the vertebrae for carcharhinid shark species that prey on animals with high levels of bioaccumulation [62, 63] implies that zinc does not bioaccumulate in shark vertebrae. Together, this suggests that bioaccumulation has a minor or negligible role in zinc deposition, and that zinc deposition is most likely primarily controlled by physiological processes.

Although the phylogenetic breadth of our study is large, the costs of synchrotron use precluded larger numbers of individuals per species. This may have restricted identification of clear patterns within and between species, especially the possibility of effects of individual age or sex. However, this first study into elasmobranch vertebrae chemistry using X-ray Fluorescence Microscopy has provided unique insights into zinc deposition in sharks and identified possible avenues for further research to understand the depositional processes within species with different developmental strategies and/or life history requirements.

## Supporting information

S1 File Raw data of signal detections for linear sections across all vertebrae examined in this study. The data also includes other elements that were detectable, but not necessarily detected in significant amounts.
(XLSX)
